# The Role of the Abdominal Perforator Exchange (APEX) Technique in the Perforator Selection Algorithm for Delayed Deep Inferior Epigastric Perforator (DIEP) Flap Breast Reconstruction

**DOI:** 10.3390/jcm14093256

**Published:** 2025-05-07

**Authors:** Dmitry V. Melnikov, Elina I. Abdeeva, Semyon I. Ivanov, Victor A. Gombolevskiy

**Affiliations:** 1 Department of Plastic Surgery, I.M. Sechenov First Moscow State Medical University, 119991 Moscow, Russia; melnikovmd@gmail.com (D.V.M.); ivanov_s_i@staff.sechenov.ru (S.I.I.); 2 Department of Reconstructive and Plastic Surgery, Lancet Clinic, 105066 Moscow, Russia; 3 Institute for Personalized Oncology, I.M. Sechenov First Moscow State Medical University, 119991 Moscow, Russia; gombolevskii@gmail.com; 4 Artificial Intelligence Research Institute (AIRI), 121170 Moscow, Russia

**Keywords:** breast reconstruction, deep inferior epigastric perforator (DIEP) flap, perforator flap, donor-site morbidity, abdominal perforator exchange (APEX) technique

## Abstract

**Background**: The deep inferior epigastric artery perforator (DIEP) flap is currently the most widely used method for autologous breast reconstruction. Its primary advantage over the transverse rectus abdominis muscle (TRAM) flap is the reduction in donor-site morbidity, as it preserves the integrity of the abdominal muscles and motor nerves. Importantly, each patient’s unique vascular anatomy requires an individualized approach to perforator selection and the surgical technique. **Objective**: We aimed to minimize donor-site morbidity and refine the perforator selection strategy in delayed DIEP flap breast reconstruction using the abdominal perforator exchange (APEX) technique. **Materials and Methods**: In this study, we prospectively and retrospectively analyzed the use of the APEX technique in patients undergoing delayed DIEP flap breast reconstruction between April 2020 and October 2024. All patients underwent preoperative non-contrast magnetic resonance angiography of the donor area. A total of 106 patients were enrolled and divided into two groups: 34 patients underwent reconstruction using the APEX technique, and 72 patients received standard DIEP flap breast reconstruction. **Results**: Our study demonstrated a statistically significant increase in operative time, averaging 30.5 min in the APEX group (*p* < 0.05). There was also a significant difference in the incidence of marginal flap necrosis between the two groups. No cases of myotomy were observed, and motor nerve transection was required in one case. **Conclusions**: The APEX technique has been shown to be reliable when standard dissection would compromise the neuromuscular anatomy of the abdominal wall without compromising perfusion in the flap.

## 1. Introduction

Currently, perforator flaps of the anterior abdominal wall are widely regarded as the method of choice for breast reconstruction (BR) due to their excellent natural esthetic outcomes compared to other techniques, greater patient satisfaction in the long-term postoperative period, and lower rates of reoperation and revision [1,2,3].

Accordingly, the deep inferior epigastric perforator (DIEP) flap is currently recognized as the “gold standard” for BR [3], having replaced the transverse rectus abdominis myocutaneous (TRAM) flap and its variations [4]. This shift is primarily attributed to the reduced donor-site morbidity achieved through microsurgical techniques and the absence of the need to include muscle in the flap to preserve its blood supply [5,6].

However, DIEP flap harvest often requires disruption to the integrity of the muscular and neural structures of the anterior abdominal wall due to the unique anatomy of the deep inferior epigastric artery (DIEA) perforator branches, their contribution to the blood supply of the rectus abdominis muscle, their complex intramuscular course, and the proximity of motor nerves [7,8,9]. As a result, surgeons are often faced with a dilemma: either sacrificing the flap’s blood supply by forgoing the dominant perforator and/or selecting the optimal number of perforators or compromising the integrity of the donor site during DIEP flap BR [9,10].

A potential solution to this issue, the abdominal perforator exchange (APEX) technique, was first proposed by Della Croce et al. in 2019 [9]. Two years later, Zoccali et al. described their clinical experience with this approach and classified the different types of DIEA bifurcation relevant to APEX [11].

This technique involves harvesting the DIEP flap through the temporary ligation of the selected perforator branches, followed by their subsequent restoration via an additional microsurgical anastomosis. This approach allows the flap to be harvested while preserving the integrity of the rectus abdominis muscles and motor nerves [9,11].

Although the reported clinical experience with APEX remains limited—with only a few authors describing its application to date—we consider this technique a promising and salvage option in select cases [9,11].

The disadvantage of this method in terms of the need for an additional anastomosis may be offset by the surgeon’s expertise and a well-coordinated workflow among the surgical team.

This study presents our clinical experience with the APEX technique and provides a comparative analysis of its outcomes versus those of the standard DIEP flap approach in delayed BR.

## 2. Objective

The objective of this study was to reduce donor-site morbidity and refine the algorithm for perforator vessel selection in delayed DIEP flap BR using the APEX technique.

## 3. Materials and Methods

In this study, we prospectively and retrospectively analyzed the results of delayed DIEP flap BR in patients who underwent surgery between April 2020 and October 2024. All surgeries were performed by a single surgical team at the Department of Plastic Surgery, Sechenov University and the Department of Reconstructive and Plastic Surgery at the Lancet clinic. This study was approved by the Local Ethical Committee of Sechenov University (Approval No. 09-23, dated 5 May 2023). Informed consent was obtained from each participant.

### 3.1. Sample Characteristics

**Figure 1:**
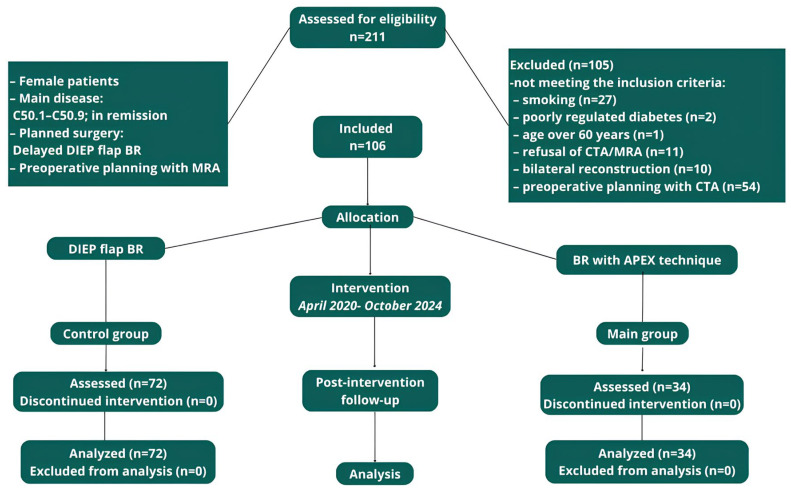
STROBE flowchart. Abbreviations: BR—breast reconstruction; MRA—magnetic resonance angiography; CTA—computed tomography angiography. Adapted from Cuschieri S [12].

A total of 106 female patients with a mean age of 45.2 (range 27–59) and a mean BMI of 28.9 (range 22–36.9) kg/m^2^, who underwent delayed unilateral BR with a DIEP flap after mastectomy, were enrolled in our study (Figure 1).

Patients were divided into 2 groups: the first included 34 women who underwent the APEX technique, and the second group included 72 patients who underwent the standard DIEP flap technique. A power analysis confirmed that this sample size achieved 80% power with 95% significance. There were no statistically significant differences in the baseline data between the two groups (*p* > 0.05 for all comparisons).

### 3.2. Assessment of Perfusion-Related Complications

Fat necrosis was evaluated using ultrasound, including color Doppler imaging, performed at 1 and 3 months postoperatively. The diagnosis was based on characteristic ultrasound features, such as hypoechoic areas ≥ 1 cm in diameter with either well-defined or ill-defined margins and decreased perfusion in the fatty tissue on Doppler imaging. In select cases, the diagnosis was further supported by clinical signs, including palpable firmness, edema, or localized inflammation.

In addition to fat necrosis, marginal flap necrosis was also systematically assessed during routine follow-up visits at 1 and 3 months postoperatively. The diagnosis was based on visual signs of superficial ischemia along the flap edges, such as discoloration, epidermolysis, or limited skin loss. When necessary, ultrasound imaging was used to clarify the depth and extent of tissue damage.

Flap necrosis was defined as partial (i.e., partial flap loss) or complete (i.e., total flap loss) tissue loss extending beyond the superficial dermal layer and typically requiring surgical intervention. Assessment was based on clinical examination, and the diagnosis was confirmed when areas of nonviable tissue failed to resolve or progressed despite conservative management.

## 4. Statistical Analysis

The statistical analysis was performed using StatTech v.4.7.3 (StatTech LLC, Kazan, Russia). All clinical and demographic variables are presented as quantitative distributions and simple percentages. The Kolmogorov–Smirnov test was used to assess the normality of the data distribution. Measures of variation, such as the standard deviation (SD), were used to characterize the dispersion of continuous variables.

The 95% confidence intervals (CIs) are reported to indicate the precision of the mean estimates. Quantitative variables that did not follow a normal distribution are described using the median (Me) and interquartile range (IQR: Q1–Q3). A *p*-value of <0.05 was considered statistically significant.

## 5. Interventions

All participants underwent preoperative planning using non-contrast magnetic resonance angiography (MRA). MRA was performed on a Philips Ingenia 1.5T scanner (Royal Philips, Best, the Netherlands). The imaging protocol was prospectively registered on ClinicalTrials.gov (PRS platform; Unique Protocol ID: NCT06061835), in accordance with established research standards.

**Figure 2:**
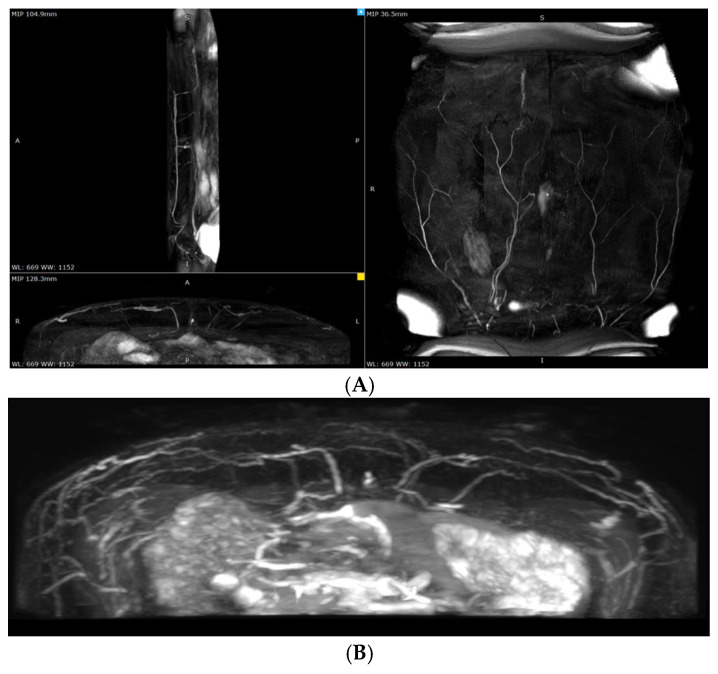
Preoperative magnetic resonance angiography scans. (**A**) Coronal plane; (**B**) axial plane.

The preliminary decision regarding the APEX technique and the dominant perforator was made during the preoperative planning phase (Figure 2).

In all cases, the preoperative selection of perforator branches and the decision to apply the APEX technique were confirmed intraoperatively.

The decision to use the APEX technique was based on the following rationale: in cases where two perforators were selected from different rows and flap harvesting would otherwise have required the transection of the intervening muscle segment. This approach allowed us to avoid conversion to a TRAM flap.

### The APEX Technique

Flap elevation began with an incision made along the lower preoperative marking, extending down to the abdominal fascia. The flap was elevated in a caudal-to-cranial direction toward the anticipated perforator location.

**Figure 3:**
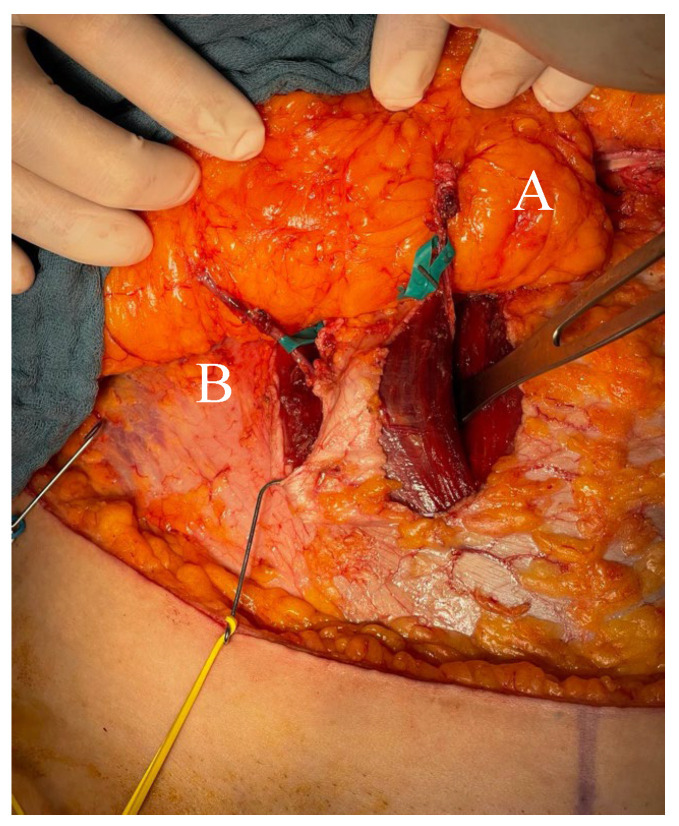
Flap harvesting stage. (***A***) Medial-row perforator; (***B***) lateral-row perforator.

**Figure 4:**
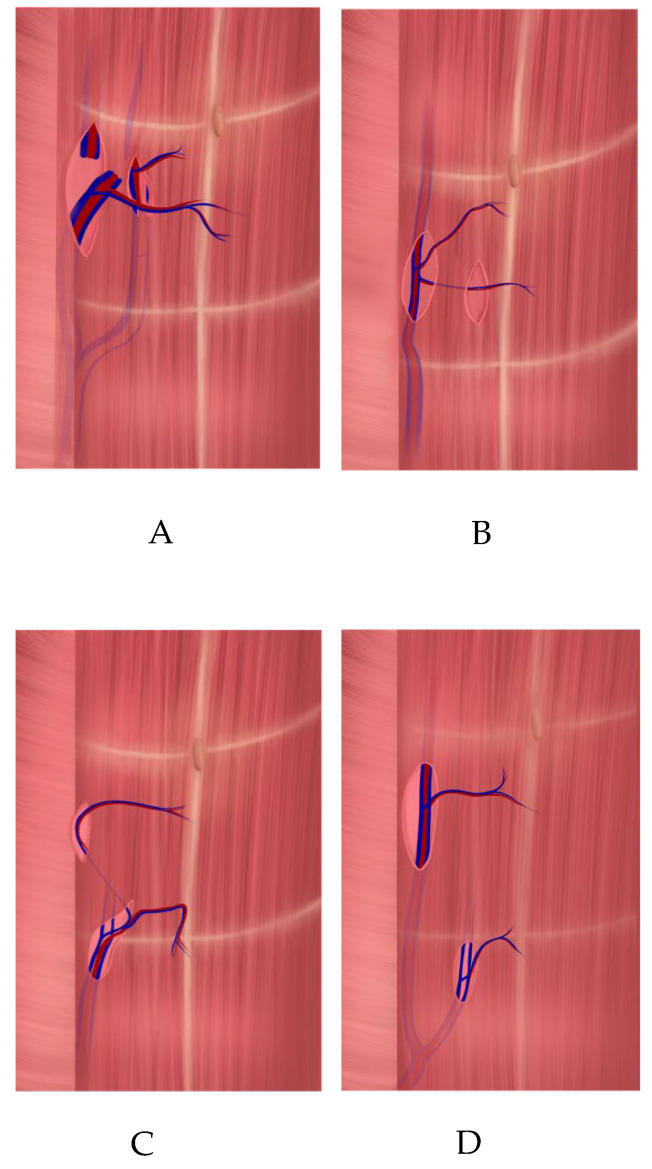
Schematic illustration of DIEA bifurcation types according to Zoccali et al. (2019): (***A***) Type A; (***B***) Type B; (***C***) Type C; (***D***) Type D.

Upon reaching the point where two perforators emerged from the fascia, dissection was continued along their course toward the DIEA. Both perforator branches of the DIEA emerged on either side of the muscle bundle (Figure 3) and joined the main pedicle either at different levels (type A, according to Zoccali) (Figure 4A) or at the same level (types B and C) (Figure 4B,C).

Beneath the muscle bundle, the medial perforator was carefully dissected free from the pedicle near the bifurcation point.

**Figure 5:**
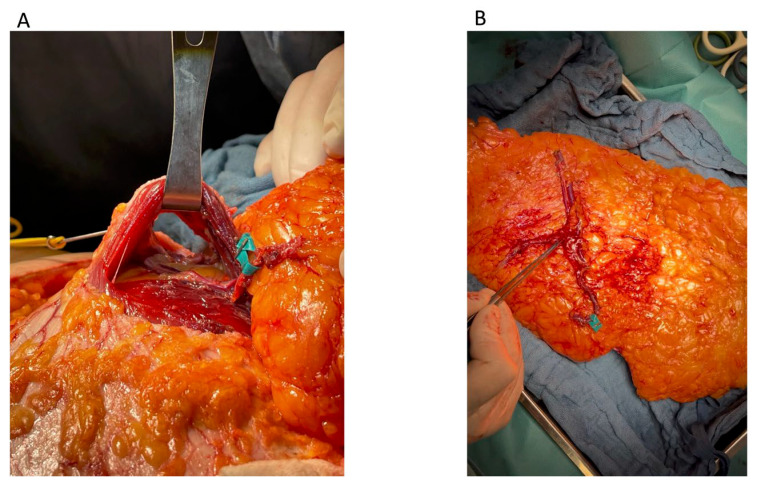
(**A**) Intraoperative view of the donor site following DIEP flap harvesting using the APEX technique; (**B**) the DIEP flap harvested using the APEX technique.

Each perforator was mobilized from its respective intramuscular plane, allowing the preservation of the muscle bundle and the motor nerves located between the two perforators (Figure 5A).

The medial perforator (Figure 5B) was then reattached to the pedicle via end-to-end microvascular anastomosis at the point of its initial transection, thereby restoring the continuity of the vascular pedicle. This step was consistently performed on a separate microvascular table during the flap preparation phase.

Subsequently, the reconstructed pedicle was anastomosed to the internal mammary vessels in the recipient site. In all cases, both arterial and venous anastomoses were performed to ensure adequate perfusion of the flap.

## 6. Results

According to the analysis of the MRA images and medical records, among patients who underwent the APEX technique (main group), type A bifurcation of the DIEA (as classified by Zoccali et al. [11]) (Figure 4A) was the most common, observed in 25 patients (23.6% of the total cohort). Type C (Figure 4C) was identified in six cases (5.6%) and type B (Figure 4B) in three cases (2.8%).

Among the four patients in the control group, type D bifurcation of the DIEA was identified (Figure 4D). However, the APEX technique was not applied in these cases due to the deep intramuscular course of the perforators and small vessel diameters. These patients underwent the standard DIEP flap procedure using perforator vessels from the contralateral side, with either one (*n* = 3) or two (*n* = 1) medial-row perforators.

### 6.1. Overall Operation Time, Pedicle Cut Time, and Flap Preparation Time

The comparative analysis showed a statistically significant increase in the overall operation time in the main group—by more than 30.5 min (*p* < 0.001). This increase was due to the longer dissection time, flap preparation time prior to transfer to the recipient site, and flap ischemia time (Table 1).

Within the main group, patients with bifurcation types B and C demonstrated longer dissection and flap preparation times compared to those with type A (Table 2).

No statistically significant differences in dissection time were observed between patients with type A bifurcation and those in the control group (*p* = 0.319).

### 6.2. Donor-Site Morbidity

The length of the fascial incision was shorter in the APEX group compared to the standard DIEP group; however, there was no statistically significant difference in the vascular pedicle length between the two groups (Table 3).

None of the patients in the APEX group required myotomy, and motor nerve transection was necessary in only one case.

All patients who underwent the standard DIEP technique required an average fascial incision length of 0.95 cm (Q1–Q3: 0.80–1.20; range: 0.40–1.80). An analysis of the impact of the perforator characteristics on the myotomy length did not reveal any statistically significant differences.

### 6.3. Venous Superdrainage and Arterial Supercharging

The decision to apply venous superdrainage and arterial supercharging using the superficial epigastric vessels was based on intraoperative perfusion assessment (capillary refill test).

A statistically significant difference in the use of arterial supercharging was observed between the groups: the rate was 16.67% (*n* = 12) in the control group (all of whom had flaps harvested on a single perforator), while, in the main group, the inclusion of the superficial epigastric artery in the flap was required in only one case (a patient with type C bifurcation).

No statistically significant differences were found between the groups regarding the use of the superficial epigastric vein for venous superdrainage (*p* = 0.920).

### 6.4. Complications

A statistically significant difference was noted in the incidence of limited marginal flap necrosis and fat necrosis between the groups (*p* < 0.05), with lower complication rates in the main group.

Additionally, an analysis of marginal necrosis within the main group showed that the type A configuration was associated with marginal necrosis in one case (4%), compared to two cases with type B (33.3%) and one case with type C (33.3%) bifurcation.

There were no statistically significant differences in the fat necrosis rates within the main group based on the anatomical configuration of the perforators (*p* = 0.313).

A comparative analysis of severe microsurgical complications did not reveal statistically significant differences between the groups (*p* = 0.294). In the APEX group, two patients developed arterial thrombosis of the vascular pedicle requiring revision: one case (2.9%) resulted in partial flap loss (less than 50% of the total flap area), and one case (2.9%) resulted in complete flap loss.

In the control group, four patients (3.8%) experienced severe perfusion-related complications requiring flap revision. Among them, one patient (1.4%) developed arterial thrombosis of the vascular pedicle with revision without flap loss. Venous thrombosis requiring revision and partial flap loss (less than 50% of the flap area) occurred in two cases (2.8%), and one patient (1.4%) experienced venous thrombosis with revision and complete flap loss.

### 6.5. Perforator Characteristics in the Control Group

A total of 112 perforator vessels were harvested in 72 patients in the control group. Of these, 32 flaps (44.4%) were raised on a single perforator, and 40 flaps (55.6%) were raised on two perforators.

Among the flaps harvested on a single perforator, 18 (25.0%) utilized a single medial-row perforator and 13 (18.1%) utilized a single lateral-row perforator.

Of the 40 flaps raised on two perforators, 26 flaps (36.1%) were based on a combination of one medial and one lateral perforator on the same side. Five flaps (6.9%) were raised on two lateral-row perforators, while nine flaps (12.5%) were based on two medial-row perforators from the contralateral side.

When flaps were harvested using two perforators, the superior perforator of the medial row was preferentially selected whenever feasible. The vascular pedicle length averaged 12.3 cm (range: 9.0–17.0).

## 7. Discussion

The current algorithm for perforator vessel and technique selection, as proposed by A. Mohan et al., includes conversion to a muscle-sparing TRAM flap in cases where two necessary perforators are located in different rows [10].

In our practice, we incorporated the APEX technique into the decision-making algorithm for such cases. In this study, we evaluated the efficacy and safety of the APEX technique compared to the standard DIEP flap harvesting approach in delayed unilateral BR.

Our findings confirm previous studies demonstrating the potential to preserve the neuromuscular structures of the anterior abdominal wall when using the APEX technique [9,11]. Moreover, our results are consistent with the conclusions drawn by Zoccali et al. [11] regarding the technical nuances and differing risk profiles associated with various DIEA bifurcation types.

A statistically significant increase in the total operative time was observed in the APEX group, with average prolongation of 30.5 min compared to the control group. This value is approximately half the increase reported by Della Croce et al., who documented a mean prolongation extent of 68.8 min for unilateral and 67.5 min for bilateral reconstruction in the APEX group [9].

Furthermore, the subgroup analysis within the APEX group revealed that the increase in operative time was primarily associated with patients presenting with bifurcation types B and C. In contrast, patients with type A bifurcation did not demonstrate a statistically significant difference in dissection time compared to the control group.

While Della Croce et al. did not detail the workflow of their surgical team, we attribute the discrepancy in the operative time to our coordinated two-team approach, which allows for the simultaneous execution of procedural steps.

The DIEP flap is widely recognized for its ability to minimize donor-site morbidity by preserving the rectus abdominis muscles and motor nerves, thus offering a key advantage over the TRAM flap [4,5]. However, accumulating evidence suggests that pedicle dissection during standard DIEP flap harvest may still compromise the integrity of neuromuscular structures at the donor site [13].

Several studies have emphasized that the use of lateral-row perforators is particularly associated with an increased risk of nerve damage and the need for myotomy, both of which can negatively impact anterior abdominal wall function [9,11,13,14].

These findings are consistent with our results, which demonstrate an association between motor nerve transection, the use of lateral-row perforators, and lower patient-reported functional outcomes. The APEX technique significantly reduced the rate of motor nerve transection (*p* < 0.001) and eliminated the need for myotomy.

In addition to the APEX technique, other strategies aimed at minimizing donor-site morbidity—such as the limited fascial incision (LFI) technique and its modifications—have also been described in the literature [15,16,17].

Our results similarly demonstrated a modest, but statistically significant, reduction in the fascial incision length (approximately 1 cm on average) with the use of the APEX technique.

However, we do not consider this to be the primary benefit of the method.

A similar approach was selectively applied in some patients in the control group, and fascial preservation was not always feasible.

However, fascial incision reduction is not always achievable. While minimizing the fascial incision may limit muscle dissection, it does not eliminate the need for myotomy or motor nerve transection [9,11].

Furthermore, while reducing the fascial incision length may reduce the extent of muscle dissection, it does not obviate the need for myotomy or nerve transection—particularly in cases involving deeply intramuscular perforators or converging branches (as seen in types B and C) [11].

Our findings suggest that the APEX technique effectively addresses these anatomical challenges.

In the APEX group, no patients required myotomy, whereas all patients in the control group underwent transverse myotomy, with an average width of 0.95 cm.

Regarding acceptable thresholds for myotomy in standard DIEP procedures, our analysis did not reveal a statistically significant correlation between the extent of myotomy and the functional outcomes of the anterior abdominal wall.

This may be attributed to the relatively small sample size and the retrospective nature of the study.

Nonetheless, we agree with the conclusion drawn by Zoccali et al. that clinical judgment and individualized surgical decision-making are essential when determining acceptable thresholds for muscle transection in DIEP flap procedures.

While Della Croce et al. highlighted the potential impact of the APEX technique on flap design and the esthetic positioning of the lower abdominal scar [9], in our practice, the flap design was consistently tailored to achieve low scar placement, regardless of the perforator configuration or surgical technique used.

The potential compromise between minimizing donor-site morbidity and optimizing flap perfusion remains a critical consideration in DIEP flap surgery. The APEX technique was developed specifically to address this challenge [10,18].

In our cohort, the use of the APEX technique was associated with a reduced incidence of marginal flap necrosis.

Notably, type A bifurcation was linked to the lowest rates of both marginal and fat necrosis.

Although no statistically significant differences in severe microsurgical complications were observed between groups, all cases of vascular pedicle thrombosis occurred in patients with type B or C bifurcation. Importantly, no patients with type A bifurcation developed perfusion-related complications or required revision surgery.

These findings further support the concept proposed by Zoccali et al., suggesting that DIEA bifurcation patterns may influence the technical feasibility of the APEX approach and should be taken into account when selecting appropriate candidates for this technique [11].

## 8. Limitations

We acknowledge several limitations of our study, primarily related to differences in patient sampling. However, the sample size and statistical methods employed were deemed appropriate for the type and scope of analysis conducted.

We also recognize that the number of patients with bifurcation types B and C in the APEX group was limited. Although appropriate statistical methods were applied, the findings related to these subgroups, particularly differences in perfusion-related complications, should be interpreted with caution. Additional data from a larger number of cases will be required to draw more definitive conclusions.

Furthermore, this study did not include systematic measurements of the arterial and venous diameters at the time of vascular anastomosis. Such data could provide valuable insights into the technical complexity associated with different APEX subtypes.

## 9. Conclusions

The APEX technique enables DIEP flap harvest using two perforator vessels with a shared dominant pedicle, while minimizing damage to the rectus abdominis muscles and motor nerves.

Incorporating the APEX technique into the perforator selection algorithm may help to avoid conversion to a TRAM flap while preserving reliable flap perfusion.

This approach appears particularly beneficial for patients with DIEA bifurcation types in which perforator branches arise at different levels.

## Figures and Tables

**Table 1:** Comparative analysis of the operation time: DIEP vs. APEX. *—statistically significant difference (*p* < 0.05).

	Overall Operation Time (min)			*p*
Group	Me	Q_1_–Q_3_	n	
DIEP	337.00	315.00–355.00	72	<0.001 *
APEX	367.50	350.00–390.00	34	
	Pedicle cut time (min)			
DIEP	36.50	29.00–45.00	72	0.005 *
APEX	41.00	35.00–55.00	34	
	Flap ischemia time (min)			
DIEP	84.00	78.00–90.00	72	<0.001 *
APEX	95.00	90.00–113.75	34	
	Flap preparation time (min)			
DIEP	30.00	27.75–39.25	72	<0.001 *
APEX	52.50	45.00–60.00	34	

**Table 2:** Analysis of the operation time: APEX group. *—statistically significant difference (*p* < 0.05).

	Overall Operation Time (min)			*p*
	Me	Q_1_–Q_3_	n	
A	355.00	340.00–370.00	25	<0.001 *
B	412.50	398.75–460.00	6	p_B–A_ < 0.001
C	410.00	400.00–427.50	3	p_C–A_ = 0.017
	Flap ischemia time (min)			
A	90.00	90.00–95.00	25	<0.001 *
B	125.00	117.50–136.25	6	p_B–A_ < 0.001
C	135.00	125.00–140.00	3	p_C–A_ = 0.007
	Flap preparation time (min)			
A	45.00	45.00–55.00	25	<0.001 *
B	76.00	67.25–79.50	6	p_B–A_ = 0.003
C	82.00	78.50–86.00	3	p_C–A_ = 0.004

**Table 3:** Comparative analysis of fascial incision and pedicle length between the two groups. *—statistically significant difference (*p* < 0.05).

Group	Length of Fascia Incision (cm)			*p*
	Me	Q_1_–Q_3_	n	
DIEP	9.30	8.40–10.35	72	0.003 *
APEX	8.30	6.50–9.80	34	
Pedicle Length (cm)				
DIEP	12.00	11.00–13.50	72	0.697
APEX	12.50	11.00–13.15	34	

## Data Availability

The raw data supporting the conclusions of this article will be made available by the authors upon request.
